# Psychological Wellbeing and Perceived Fatigue in Competitive Athletes after SARS-CoV-2 Infection 2 Years after Pandemic Start: Practical Indications

**DOI:** 10.3390/jfmk8010001

**Published:** 2022-12-20

**Authors:** Andrea Buonsenso, Arianna Murri, Marco Centorbi, Giulia Di Martino, Giuseppe Calcagno, Alessandra di Cagno, Giovanni Fiorilli, Enzo Iuliano

**Affiliations:** 1 Department of Medicine and Health Sciences, University of Molise, v. De Sanctis 1, 86100 Campobasso, Italy; 2 Department of Motor, Human and Health Sciences, University of Rome “Foro Italico”, Lauro de Bosis Square 15, 00197 Rome, Italy; 3 Department of Theoretical and Applied Sciences, eCampus University, 22060 Novedrate, Italy

**Keywords:** COVID-19 pandemic, survey, return to play, symptoms, health-related quality of life

## Abstract

The COVID-19 pandemic deeply affected sports and athletes, influencing performance and psychological wellbeing. In order to provide useful guidelines for coaches, a web-based survey was conducted. Three web-based questionnaires were administered during the last phase of the Omicron wave to a total of 204 Italian athletes (age 24.96 ± 9.82): an informative questionnaire to collect sociodemographic data and infection symptoms information, the Fatigue Severity Scale (FSS) and the General Health Questionnaire-12 (GHQ-12). No differences between infection sequels of different variant typologies were found over the long term after the infection. The most frequently declared symptoms included cough (50%), muscular skeletal impairments (48%) fatigue (43%) and fever (43%). Results showed that female athletes have a higher risk of developing post-COVID-19 symptoms, GHQ-12 worse results (*p* = 0.005) and greater fatigue (*p* = 0.0002) than males. No significant difference in infection incidence between high- and low-level athletes was found. Endurance athletes showed greater perceived fatigue than anaerobic sports athletes (*p* = 0.045). Conclusions: These results suggested the need for specific approaches and continuous updating to differentiate training programs for different athletes during the return to play. Medical controls and daily monitoring of athletes of all levels after the infection could be advisable.

## 1. Introduction

Two years of the SARS-CoV-2 pandemic deeply influenced sports and athletes [1]. Before the pandemic, in Italy, 20% of adults, 75% of children and 59% of adolescents practiced sports activities, whereas, after this period, 25% of adults, 48% of children and 30% of adolescents dropped out; among the youngest athletes, one in three has changed sport [2]. Regarding competitive athletes, the first period of the pandemic quarantine impacted the planning and scheduling of athletes’ routines [3]. Canceled or postponed events and limited access to facilities affected both performance and psychological aspects, increasing distress [4,5]. The problems linked to the pandemic restrictions involved both athletes worried about their sport career concerns and managers for financial aspects [6]. Successively, despite the highest transmissibility of omicron variants, athletes have resumed their usual rhythms of training and competitions, and the interruptions due to COVID-19 infection were individually managed as a break due to a normal individual illness [3]. Omicron variants’ consequences were less serious than previous variants being attenuated by previous widespread infections and vaccinations [7,8]. Nevertheless, the increasing number of infections and re-infections among the general population and athletes [9] induced several difficulties in the regular aspects of sport life, with severe impact on physiological and psychological aspects, with negative repercussions on performance and health [3,10].

The clinical manifestation of almost all COVID-19 variants among athletes appears to be asymptomatic or have mild symptoms; nevertheless, recent reports highlighted that asymptomatic or mildly symptomatic disease manifestations could hide serious underlying pathologies and several complications that must be carefully monitored [11]. Nevertheless, the severity of the acute phase of the illness comes with a risk of continued post-COVID symptomatology [12]. The so-called “Long-COVID”, defined by the World Health Organization, is a condition of persistent signs and symptoms over time after the acute phase, which affects athletic recovery [13]. Fatigue, dyspnea, muscle soreness, chest pain, difficulty concentrating, anxiety and depression may affect athletes’ performance for many weeks after the end of the infection period, even after several months [14]. In athletes, fatigue entails a decrease in physical performance, due to an internal homeostasis breakdown, with a decrease in neuromuscular strength and accumulation of waste metabolites with an enhancement of the perceived difficulty in performing their training tasks [15]. Consequently, appropriate restart after isolation and specific rules for “return-to-play” protocols are needed and must be constantly updated.

Nowadays, the return-to-play protocols are constantly changing and aim only to help athletes and teams stay safe in recovering their performance [16]. The return to training and competition after COVID-19 still represents a huge challenge for sports medicine teams and coaches that are responsible for the management of athletes. In addition to the constrained interruption of training routines that determines a detraining condition, the presence of lower respiratory tract features and fatigue reduces athletic performance, requiring an adjustment in the training planning [17]. Moreover, early return prior to full recovery represents a risk factor, and close medical monitoring after infection is recommended [18].

It is important to increase knowledge about the response to COVID-19 infection based on the peculiar characteristics of sports discipline, competition level, gender and age in order to apply different approaches that are case-specific [19].

This web-based survey aimed to first assess the relationship between sports disciplines, competitive levels, age, gender and susceptibility to COVID-19, considering the association of different SARS-CoV-2 symptoms and their consequences on the training resume. Moreover, quality of life after the infection and fatigue perception during the return to play was investigated. We analyzed the differences in side effects during and after the disease, and between different waves of COVID-19 and the side effects during and after illness that could have influenced the athletes’ return to play.

The results of the survey may provide knowledge for sports physicians, kinesiologists and coaches to better understand the course of COVID-19 among different athletes to individualize the planning and scheduling of sports recovery.

## 2. Materials and Methods

### 2.1. Study Design

This study is a retrospective cohort study trying to investigate whether factors such as gender, sport type, metabolic demand, technical level, virus variant, severity of the infection and type of symptoms may influence the fatigue and the general health perception after SARS-CoV-2 infection in a population of competitive athletes.

### 2.2. Participants

Participants were asked to complete an online survey, and they were recruited throughout the Italian country using an online snowball sampling strategy. A total of 204 self-selected athletes (age 24.96 ± 9.82 years old) were included in this study. The minimum sample size was calculated a priori using G*Power software (v.3.1.9.6). The analysis indicated that a minimum sample size of 148 participants was necessary to have a moderate effect size *f* = 0.30. The inclusion criteria were: (1) have been positive for SARS-CoV-2 and successively becoming negative in the last two months from the date of completion of the survey, (2) affiliated with a National Federation and/or a sports association from at least 2 years, (3) maintain a healthy and active lifestyle in the 2 last years and (4) have no chronic or temporary respiratory, metabolic or cardiovascular diseases prior to SARS-CoV-2 infection and have not suffered important injuries and/or major surgery in the last year. The survey was preceded by a cover letter informing about the nature of the study and assuring confidentiality and anonymity. Electronic informed consent was obtained from all participants by e-mail. For underage participants, informed consent was obtained from parents or guardians who were required to administer the questionnaire on behalf of their son/daughter. The study was designed and conducted in accordance with the Declaration of Helsinki and approved by the Scientific Technical Committee of the Department of Medicine and Health Sciences, University of Molise (Prot. n. 05/2022).

### 2.3. Procedures

The survey was administered during the last phase of the Omicron wave of COVID-19 (1 November 2021 to 30 April 2022) using an online platform (Google Form, Google, Mountain View, CA, USA).

The first section of the survey aimed to collect sociodemographic data and information on the sport practiced, age, gender, sport type (individual or team sports), main metabolic demands (aerobic, anaerobic or mixed sport) and technical level (elite or sub-elite level). Main metabolic demands were established on the basis of the classification proposed by Dal Monte [20]. Concerning the technical level, athletes were considered elite when they represented their sport at major competitions in international contests, while sub-elite athletes were those who competed at national or regional sports events.

The second section aimed to assess which virus variant was contracted (delta or omicron), the severity of the infection (slight, moderate or severe), the type of symptoms that the participants experienced during the SARS-CoV-2 infection (multiple choice was allowed for this question), the type of symptoms during the return to play after the SARS-CoV-2 infection (multiple choice was allowed for this question), and eventual complications after recovery. The severity was established on the basis of the following criteria: participants with no symptoms and participants who did not seek medical intervention were considered as slight severity; participants who required medical intervention without hospitalization were considered as moderate severity; finally, participants who were hospitalized were instead considered to be severe cases. This second section of the survey is shown in Supplementary Materials (Document S1: Second section of the survey).

The information collected in these first two sections was used to divide the sample into different categories and groups successively used for statistical analysis.

In the third section, the Fatigue Severity Scale (FSS) [21] and the General Health Questionnaire-12 (GHQ-12) [22] were administered to assess conditions reported in the return to play by athletes after the virus infection.

***Fatigue Severity Scale (FSS):*** This questionnaire includes 9 items, each one with a Likert rating scale from 1 (strongly disagree) to 7 (strongly agree). Each item is a statement on fatigue, investigating its relationship with work, motivation, physical activity and social life. The total score, which ranges between 9 and 63, assesses the impact and severity of fatigue on daily living. A Total Score (TS) greater than 35 (cut-off) indicates a critical level of fatigue and suggests further evaluation by a physician [23].

***General Health Questionnaire-12 (GHQ-12):*** The GHQ-12 is a short version of the questionnaire used as a screening to identify psychological distress in the general population. Specifically, the GHQ allows us to investigate the presence of four elements of distress: depression, anxiety, social deterioration and hypochondria (indicated by somatic symptoms). The questionnaire consists of 12 items, each one with a 4-point Likert scale: 0 (not at all), 1 (same as usual), 2 (rather more than usual) and 3 (much more than usual). The total score ranged from 0 to 36, with higher scores representing higher levels of distress [24]. The total score was evaluated as follows: normal (score from 0 to 14); mild (score from 15 to 19); severe (score from 19 to 36).

### 2.4. Statistical Analysis

Firstly, a descriptive analysis was performed. The median and the interquartile range (IQR) of the variables age, FFS and GHQ-12 scores were computed both for all the participants together and for different groups separately. These groups were stated according to the answers of the first two sections of the survey (described in the next paragraph). The Kolmogorov–Smirnov test was used to assess normality. Median and IQR were used for mean and standard deviation due to the not normal distribution of all 3 variables.

Then, comparative analyses were performed to evaluate significant differences among the groups in the variables age, FFS and GHQ-12 scores. The non-parametric Kruskal–Wallis *H* test was used to evaluate significant differences in gender (males vs. females), sport type (individual vs. team sport), metabolic demand (aerobic vs. anaerobic vs. mixed aerobic-anaerobic), technical level (elite vs. sub-elite level), SARS-CoV-2 variants (delta vs. omicron) and severity of the infection (slight vs. moderate vs. severe). When the category included more than 2 groups and the Kruskal–Wallis *H* test indicated a significant difference among these groups. The Mann–Whitney *U* test was used for pairwise comparisons utilizing the Bonferroni correction for multiple comparisons.

Successively, stepwise multiple regression analyses were used to evaluate whether the presence of one or more symptoms during the SARS-CoV-2 infection or during the return to play could be correlated with a higher score in FSS or GHQ-12. For this analysis, the presence/absence of each symptom, declared by the participants in the second section of the survey, was codified as an independent dummy variable. In particular, the following variables have been considered for the analyses of the symptoms that occurred during the SARS-CoV-2 infection: cough, expectorate, dyspnea, fatigue, sore throat, respiratory failure, chest pain, muscle pain, joint pain, muscle fatigue, headache, taste or smell alteration, abdominal pain, diarrhea, ophthalmic symptoms, fever. The following variables have been considered for the analyses of the symptoms that occurred during the return to play after the SARS-CoV-2 infection: cough, dyspnea, chest pain, muscle fatigue, joint pain, dizziness, headache, agitation and arrhythmias. The symptoms reported by less than 4 people (2% of the sample) were not considered because the small number of cases did not allow reliable analyses.

Finally, all the symptoms that occurred during the SARS-CoV-2 infection or during the return to play were described using upset plots in order to graphically show the number of participants that reported a specific type of symptom (set size of the plot) and the number of participants that reported a specific combination of symptoms (interaction size of the plot). Due to their elevated number, the symptoms were grouped according to the organs or systems affected.

Data analysis was performed using the statistics software SPSS version 26.0 (IBM, Chicago, IL, USA). The α level was set to 0.05.

## 3. Results

The medians and the IQRs of age, FFS and GHQ-12 scores are reported in Table 1. In the same table, we report the full results of the comparative analyses that showed significant differences between gender for age (*H_(1)_* = 4.297; *p* = 0.038), FFS (*H_(1)_* = 14.367; *p* = 0.0002) and GHQ-12 scores (*H_(1)_* = 7.946; *p* = 0.005) with better scores for males in both the 2 survey despite their significant older age; significant differences were found also for sport metabolic demand for age (*H_(2)_* = 10.779; *p* = 0.005), and FFS (*H_(2)_* = 6.778; *p* = 0.034) with post hoc indicating better score in FSS for athletes involved in anaerobic sports compared with those involved aerobic sports (*p* = 0.045); significant differences were found also for the severity of the infection for FFS (*H_(2)_* = 12.655; *p* = 0.002) and GHQ-12 scores (*H_(2)_* = 8.430; *p* = 0.015) with post hoc indicating better score in FSS for participants with a slight severity of the disease compared with participants that experienced moderate and severe symptoms (*p* < 0.05), and a GHQ-12 significantly higher in participants with more severe symptoms compared to those with moderate or slight symptoms (*p* < 0.05). No differences were found for the comparisons between individual vs. team sport, elite vs. sub-elite level and between the variants delta vs. omicron.

The results of the stepwise regression analysis showed that, during the SARS-CoV-2 infection, the concomitant presence of dyspnea, fatigue, and respiratory failure are significantly associated with higher scores in FSS with a moderate correlation (*p* < 0.001; *r* = 0.463), while the concomitant presence of respiratory failure, abdominal pain and joint pain are significantly and moderately associated with higher scores in GHQ-12 (*p* < 0.001; *r* = 0.422). Concerning the symptoms experienced during the return to play, dyspnea, joint pain and dizziness are significantly and moderately associated with higher scores in FSS (*p* < 0.001; *r* = 0.594), while cough, muscle fatigue, agitation and dyspnea are significantly and moderately associated with higher scores in GHQ-12 (*p* < 0.001; *r* = 0.495). The other considered symptoms showed weak correlations both with FSS and GHQ-12 and, consequently, were not considered in the stepwise regression analyses. Detailed results are reported in Table 2.

**Figure 1:**
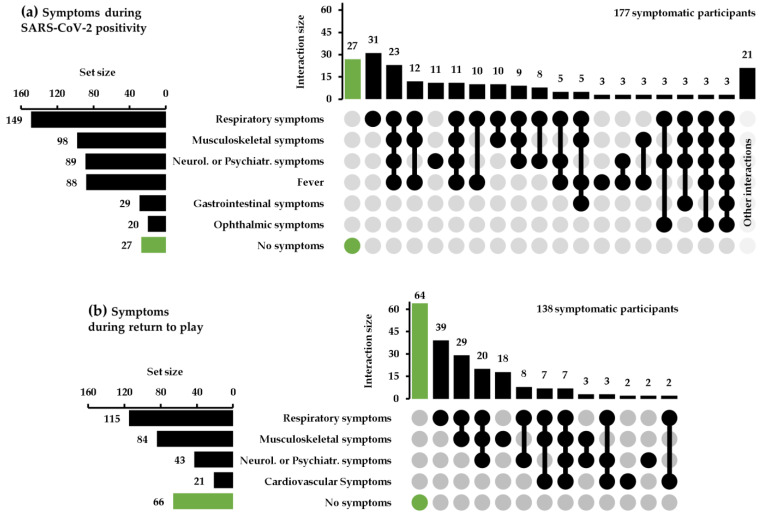
Upset plot of the symptoms experienced by the participants: (**a**) symptoms experienced by participants during the period of SARS-CoV-2 infection; (**b**) symptoms experienced by participants after the SARS-CoV-2 infection when participants returned to play.

Finally, in Figure 1, the upset plots showed the distribution of the symptoms experienced by the participants of the present study.

## 4. Discussion

This study aimed to evaluate distress symptoms that influence the quality of life and perceived fatigue in competitive athletes after one month or more from COVID-19 infection to provide suggestions for updating strategies to return to exercise.

The findings of this survey recorded a low percentage of athletes (2.5%) who experienced severe infection, with no differences between male and female athletes, while most of them declared to have had moderate (55.4%) to mild (42.2%) symptoms after the infection. Early reports suggested that even people who were asymptomatic or mildly symptomatic could have serious underlying pathology [25]. Considering that this sample consisted of young individuals and well-trained athletes, even though infected by SARS-CoV-2, generally, their symptoms, from mild to moderate, commonly involved the upper respiratory tracts, as was shown in previous studies [26]. Physical activity and training have been shown to reduce the severity of respiratory symptomatology [27], playing a role in enhancing the circulating lymphocytes and cortisol that stimulate the activity of the anti-inflammatory cytokine [28]. As well as in the general population, in our sample, the severity of symptomatology was higher in older participants [29].

The most frequently declared symptoms during COVID-19 infection included cough (50%), muscular skeletal impairments (48%), fatigue (43%), fever (43%), gastrointestinal issues (14%) and ophthalmic issues (10%). Cardiac involvement and diagnostic criteria for myocarditis concerned only three participants (1.5%), in line with previous data on young athletes after COVID-19 disease, with a heterogeneous prevalence in the range 0–15% [30]. Although we know the serious consequences of myocarditis for an athlete [31,32], until now, the relationship between SARS-CoV-2 and myocarditis is not well established. However, we advise the careful monitoring of athletes in their return to play [33]. Gastrointestinal symptoms are mainly associated with severe disease in females and elderly patients [34]. Nevertheless, in our sample, no athlete felt these symptoms during the return to play. Although knowing that SARS-CoV-2 transmission could be mediated by eye secretions, the associations between SARS-CoV-2 and ophthalmic manifestations are not proven [35]. In our sample, this manifestation involved only a small group of athletes in the early stage of the disease and nothing during the return to play. It is expected that skeletal muscle-related symptoms are common in athletes both in acute and post-acute infection [36].

Long term-symptoms related to SARS-CoV-2 infection, a broad range of symptoms that persist beyond the acute phase of COVID-19, were heterogeneous and affected different systems, as current evidence confirms [37]. Persistent symptoms of fatigue, anxiety and dyspnoea were the most common symptoms in chronic post-COVID syndrome, with unclear etiology. Age, gender and a greater number of COVID-19 symptoms at the acute phase represent risk factors associated with post-COVID symptoms [38].

A previous study indicated a possible relationship between long symptoms after SARS-CoV-2 infections and the anti-inflammatory response pathways and mitochondrial stress, even after 40–60 days post-viral infection and in asymptomatic and moderately affected patients [39]. The combination of the cytokine storm and CNS entry of the virus can cause neuroinflammation, which can lead to prolonged generalized symptoms, including fatigue, headache, myalgias and dyspnoea [40].

The results of this study on the prevalence and distribution of COVID-19 variants showed that the Omicron variant was the prevalent infection in our sample, underlining that the increased incidence may be due to greater omicron transmissibility. In fact, the return to play has raised the number of contacts and the laxity in compliance with measures to counteract COVID-19 transmission has enhanced its transmissibility [7]. Nevertheless, no differences between SARS-CoV-2 infection sequels, caused by different variant typologies, were found in distress and fatigue perception over the long term after the infection. Consequently, athletes who showed asymptomatic or low/moderate symptomatology, independently by the typology of the SARS-CoV-2 variant, may be treated in the same manner in terms of training planning, loads and rest/ratio during the return to play.

Although men and women exhibit the same probability of being infected by SARS-CoV-2 [41], female athletes showed a higher risk of being prone to post-COVID syndrome [42]. Our results as well confirmed that female athletes are more vulnerable to developing post-COVID symptoms. The findings of this survey recorded a significant gender difference in both psychological distresses, assessed by GHQ-12, and fatigue perception, assessed by FSS. Female athletes showed worse GHQ-12 results than males after COVID-19 infection. Considering the higher level of distress for females, the female sex may be considered a risk factor for the development of some long-term post-COVID symptoms such as fatigue and mood disorders [43]. As well as in the general population, female athletes, being more susceptible to experiencing distress, differed from males with regard to both adaptive and reactive behaviors to stressful events due to a combination of physiological and psychological aspects [44]. Probably, women may potentially feel the SARS-CoV-2 infection consequences more seriously and consequently are more worried than men [45]. Subjective perception of fatigue is greater in women, probably due to different interpretations of effort and stress. Regarding sports, fatigue perception is greater in women than men, even if as a result of the same load workout. Despite the fact that women have a higher efficiency in lipid oxidation, they showed a lower efficiency in the anaerobic lactic pathways and, therefore, are less efficient in rapid tests than men [46]. As a result, gender differences should be considered when managing athletes in their return to exercise, underlining that specific training interventions should be designed especially for them. It should also be considered that a safe and early return to competition for female athletes is particularly relevant because, as demonstrated in a recent review, women’s sport was the most negatively affected during the SARS-CoV-2 pandemic compared to men’s [47].

Although the most common long-COVID symptom is fatigue [48], the findings of this survey recorded a moderate correlation between GHQ-12 and fatigue (*r* = 0.398) in all athletes. Interestingly, the symptoms of fatigue affect GHQ-12 for only 15 % (*r^2^* = 0.15), indicating that it is not the main factor affecting the perception of their overall health. Frequently, recovery after COVID-19 infection is slow and incomplete [49]. In line with the National Institute for Health Research [50], these data suggest that long-COVID symptoms could be akin to an overtraining syndrome, such as an imbalance of physical, hormonal, and mental-emotional stress in an athlete’s life. Athlete recovery strategies used in overtraining syndrome could also be suggested in long COVID recovery.

Regular physical activity improves the immune system; however, the exercise characteristics, such as high frequency and intensity, long duration and type of exercise, influence this effect [27]. Indeed, elite athletes, who perform high-intensity training, could be more likely to have impaired immune system function and contract infections [51]. However, in this sample, no significant difference in infection incidence between high- and low-level athletes was found. It is likely that elite athletes, being even more sensitive to infection, are supervised daily by their sports support team, including coaches and medicine team members, and this condition may reduce this risk [52,53]. Therefore, medical controls and daily monitoring of athletes of all levels after the infection could be advisable.

The majority of the athletes (58%) declared that they suffered from distress symptoms and low health-related quality of life, despite no significant difference between athletes of high and low technical levels on fatigue and GHQ-12 score. In several athletes, the persistence of the symptoms could have forced them to decrease their planned training hours, forcing them into social isolation [54]. As a result, it is likely that the persistence of the long-COVID symptoms may have enhanced distress and depression.

Concerning sports metabolic demands, endurance athletes had declared to be more fatigued than athletes of sports in which anaerobic energy production plays an essential role. Endurance athletes are more susceptible to central-cognitive fatigue, in addition to physical fatigue, which may reduce their sports effectiveness [55]. Moreover, fatigue due to COVID-19 infection requires more effort and is unlikely to be sustainable by endurance athletes [56]. Otherwise, the short duration and the rest/effort ratio of anaerobic sports induce mainly peripheral fatigue, and consequently, mental aspects may not be predominant in influencing performance [57].

Taking into consideration the results of this study, a specific approach to organizing the training program, with appropriate reconditioning strategies, is crucial for recovery after COVID-19 infection. The results indicated that athletes who showed asymptomatic or low/moderate symptomatology, independently by the typology of the SARS-CoV-2 variant, may be treated in the same manner in terms of training planning, loads and rest/ratio during the return to play. However, the latest recommendations [25] on the management of athletes during infection suggested that all athletes should not completely stop training during post COVID period, as sudden inactivity can weaken the immune system. Thus, athletes with severe physiological conditions should engage in low-intensity resistance training and supplement protein to counteract disease-induced protein. For athletes with minor symptoms, is recommended aerobic with moderate-intensity training [25]. This last recommendation is particularly relevant because it can help in maintaining adequate cardiovascular fitness since a previous study indicated that domestic confinement due to COVID-19 negatively affected cardiorespiratory parameters in active individuals [58].

Differentiated training programs should instead be developed for male and female athletes in the return to play because females should have longer recovery times and less intense training. Similarly, differentiated training programs should be developed for endurance athletes that are more susceptible to mental fatigue: it is hypothesized that the return to play strategy for these athletes should consider a decrease in cognitive load, providing feedback to increase motivation, while peripheral fatigue, which characterizes short duration performances, should be treated by looking at mechanisms underlying muscle contractile action and its adjustment; however, this indication should be confirmed by further specific studies.

Finally, this study is not without limitations. The first limitation is related to the classification of the symptoms that was based on the answers to the survey. Consequently, it is possible that participants, especially those with a slight severity, who have not needed medical intervention, might have unintentionally miscategorized their symptoms (e.g., shortness of breath can be considered a respiratory symptom but also a cardiological one). Furthermore, this study did not investigate lifestyle, nutritional or psychological aspects, but these variables may have influenced the symptoms reported by the participants. Consequently, further studies should take into account all these potential variables. Furthermore, the generalization of the results of the present study should be performed with caution due to the possible influences of the aforementioned variables.

## 5. Conclusions

Guidelines on return to sports following SARS-CoV-2 infection are essential to ensure the health of the athletes and the training effectiveness. Considering that the different COVID-19 waves showed rapid changes in transmissibility and associated symptoms, the approach to return to exercise requires continuous updating. Knowing that the interruption of training is detrimental to athletes both during illness because the sudden inactivity can further weaken the immune system and in the usual management of athletes for detraining, it is recommended during infection to engage the athletes in low-intensity resistance training and for athletes with minor symptoms is recommended aerobic exercise at moderate intensity [25], complying with an interval training regime that better allows managing fatigue.

## Figures and Tables

**Table 1:** GHQ-12 and FSS results.

Categories	Groups	n	Age (Years)	GHQ-12 Score	FSS Score
			Median (IQR)	Median (IQR)	Median (IQR)
**Gender**	Males	111	24 (21–28) ^1^	17 (15–18) ^1^	25 (15.5–35) ^1^
	Females	93	22 (17–27) ^1^	17 (16–20) ^1^	34 (22–41) ^1^
**Sport type**	Individual	106	23 (18–27)	17 (15–19)	27 (17.25–39)
	Team	98	24 (21–28)	17 (15–19)	28 (19–37)
**Metabolic** **demands**	Aerobic	48	23 (21–31) ^2^	17 (16–19.25)	35 (21.75–39) ^3^
	Anaerobic	95	24 (21–28)	17 (15–19)	26 (17–33) ^3^
	Mixed	61	21 (16–26) ^2^	17 (15–19)	30 (19–40)
**Technical Level**	Elite	96	24 (19–29)	17 (15–19)	30.5 (19–40.25)
	Sub-elite	108	23 (20–27)	17 (15–19)	27 (18–35.25)
**COVID-19** **variant**	Delta	75	24 (19.5–27)	17 (15–19.5)	30 (16.5–39.5)
	Omicron	129	23 (20–28)	17 (15–19)	27 (19–38)
**Severity of** **infection**	Slight	86	22 (17–26)	17 (15–18) ^4^	26 (15–34) ^4,6^
	Moderate	113	24 (21–28)	17 (15–19) ^5^	31 (19–40) ^6^
	Severe	5	29 (25–57)	21 (20–26) ^4,5^	45 (35–47) ^4^
**Total number of** **participants**		204	23 (20–28)	17 (15–19)	27.5 (18–38)

^1^ = significant differences males vs. females; ^2^ = significant differences Aerobic vs. Mixed; ^3^ = significant differences Aerobic vs. Anaerobic; ^4^ = significant differences Slight vs. Severe; ^5^ = significant differences Moderate vs. Severe; ^6^ = significant differences Slight vs. Moderate. IQR = Interquartile range presented as the value of the 1st quartile and value of the 3rd quartile, respectively.

**Table 2:** Results of the stepwise multiple regression analyses.

Analysis	Dependent Variable	Significant Independent Variables Included by Analysis	*r* and Adjusted *r*^2^ Values	*F*-Value and *p*-Value
FSS score vs. symptoms that occurred during the infection	FSS score	Dyspnea, Fatigue, Respiratory failure	*r* = 0.463 *r^2^* = 0.202	*F* = 18.148 *p* < 0.001
GHQ-12 score vs. symptoms that occurred during the infection	GHQ-12 score	Respiratory failure, Abdominal pain, Joint pain	*r* = 0.422 *r^2^* = 0.165	*F* = 14.418 *p* < 0.001
FSS score vs. symptoms that occurred in return to play after infection	FSS score	Dyspnea, Joint pain, Dizziness	*r* = 0.594 *r^2^* = 0.340	*F* = 27.129 *p* < 0.001
GHQ-12 score vs. symptoms that occurred in return to play after infection	GHQ-12 score	Cough, Muscle fatigue, Agitation, Dyspnea	*r* = 0.495 *r^2^* = 0.230	*F* = 16.133 *p* < 0.001

## Data Availability

The data will be shared on reasonable request to the corresponding author.

## Supplementary Materials

The following supporting information can be downloaded at: https://www.mdpi.com/article/10.3390/jfmk8010001/s1, Document S1: Second section of the survey.
