# Supplementary material for: Psychological Wellbeing and Perceived Fatigue in Competitive Athletes after SARS-CoV-2 Infection 2 Years after Pandemic Start: Practical Indications
Source: J Funct Morphol Kinesiol. 2022 Dec 20;8(1):1. doi: 10.3390/jfmk8010001 (PMC9844459; doi:10.3390/jfmk8010001)
Supplement: Supplementary file 1 [file jfmk-08-00001-s001.zip › jfmk-2073187-supplementary.pdf]

Journal of
*Functional Morphology and Kinesiology*

MDPI

**Document S1: Second section of the survey**

For an easier interpretation of the results the second section of the survey proposed in the present study was reported below.

1. Have you tested positive and then come back negative for COVID-19 in the last two months?
    - Yes
    - No
2. Which covid variant did you contract?
    - Delta
    - Omicron
    - Another variant
    - I don't know
3. How severe was your COVID-19 infection?
    - Slight: asymptomatic infection or Symptomatic infection that did not require medical attention
    - Moderate: symptomatic infection that required medical attention
    - Severe: symptomatic infection that required hospitalization
4. Did you experience any of the following symptoms during the infection? (Please indicate all your symptoms)
    - Fever
    - Respiratory symptoms
        - Cough
        - Sputum
        - Fatigue
        - Sore throat
        - Chest pain
        - Shortness of breath (dyspnea)
        - Respiratory failure
        - Other respiratory symptoms (please indicate)
    - Musculoskeletal symptoms
        - Muscle pain
        - Joint pain
        - Muscle fatigue
        - Other musculoskeletal symptoms (please indicate)
    - Neurological/psychiatric symptoms
        - Headache
        - Loss of taste or smell
        - Dizziness
        - Agitation or confusion
        - Other Neurological or psychiatric symptoms (please indicate)

- Gastrointestinal symptoms
    - Abdominal pain
    - Diarrhea
    - Vomiting or nausea
    - Other gastrointestinal symptoms (please indicate)
- Ophthalmic symptoms
    - Sore eyes
    - Red eyes
    - Other ophthalmic symptoms (please indicate)
- Cardiovascular symptoms
    - Arrhythmias
    - Myocarditis
    - Other cardiovascular symptoms (please indicate)
- Other types of symptoms
    - Please indicate

5. Did you experience any of the following symptoms during the return to play? (Please indicate all your symptoms)
    - Fever
    - Respiratory symptoms
        - Cough
        - Sputum
        - Fatigue
        - Sore throat
        - Chest pain
        - Shortness of breath (dyspnea)
        - Respiratory failure
        - Other respiratory symptoms (please indicate)
    - Musculoskeletal symptoms
        - Muscle pain
        - Joint pain
        - Muscle fatigue
        - Other musculoskeletal symptoms (please indicate)
    - Neurological/psychiatric symptoms
        - Headache
        - Loss of taste or smell
        - Dizziness
        - Agitation or confusion
        - Other Neurological or psychiatric symptoms (please indicate)
    - Gastrointestinal symptoms

    - Abdominal pain
    - Diarrhea
    - Vomiting or nausea
    - Other gastrointestinal symptoms (please indicate)
- Ophthalmic symptoms
    - Sore eyes
    - Red eyes
    - Other ophthalmic symptoms (please indicate)
- Cardiovascular symptoms
    - Arrhythmias
    - Myocarditis
    - Other cardiovascular symptoms (please indicate)
- Other types of symptoms
    - Please indicate
